# Use of advanced technologies by occupational physicians in Brazil: a descriptive study

**DOI:** 10.47626/1679-4435-2026-1528

**Published:** 2026-06-15

**Authors:** Alberto Jose Niituma Ogata, Ana Maria Malik, Evandro Penteado Villar Felix

**Affiliations:** 1 Centro de Pesquisa em Administração em Saúde, Escola de Administração de São Paulo, Fundação Getúlio Vargas, São Paulo, SP, Brazil

**Keywords:** occupational medicine, digital health, artificial intelligence.

## Abstract

**Introduction:**

Advanced technologies, especially artificial intelligence (AI), have been progressively incorporated into clinical practice. However, their adoption in occupational medicine still faces specific challenges.

**Objectives:**

To analyze the perceptions of Brazilian occupational physicians regarding the use of technologies in their daily professional practice.

**Methods:**

Observational, cross-sectional, quantitative study. A structured questionnaire was administered to a non-probabilistic convenience sample based on voluntary participation, consisting of physicians invited by email and through the communication channels of the Brazilian National Association of Occupational Medicine from May to June 2025.

**Results:**

Of the 1,600 physicians invited, 260 responded to the questionnaire (90% confidence level; 4.69% margin of error) between May and June 2025. Participants identified epidemiological data analysis (78%) and the automation of reports and medical evaluations (67%) as the areas with the greatest potential for AI application.

**Conclusions:**

There is broad potential for the use of AI in occupational health, including leave management, identification of risk factors for chronic diseases, and health promotion. Its effective implementation will require professional training and capacity building.

## INTRODUCTION

Advanced digital technologies, especially artificial intelligence (AI), have been progressively incorporated into clinical practice across different medical specialties, with applications including clinical decision support, analysis of large data volumes, automation of health care processes, and continuous health condition monitoring [^1^,^2^].

However, in occupational medicine, the incorporation of these technologies presents additional specificities related to causal nexus assessment, leave management, epidemiological analysis of working populations, and coordination with multiple institutional stakeholders.

Occupational physicians have increasingly integrated different technologies into professional practice, including electronic health records (EHRs), telemedicine platforms, wearable devices, AI, and digital health tools, aiming to improve clinical care, workplace safety monitoring, and population health management [^3^].

Wearable devices represent a rapidly expanding technological area in occupational health, particularly in the prevention of work-related musculoskeletal disorders through assessment of biomechanical risk. These technologies include sensor systems based on inertial measurement units, electromyography, pressure sensors, and optoelectronic devices used to monitor body posture, movement, and physical load in real time. Compared with traditional observational methods, wearable devices enable continuous measurements of multiple body segments with greater precision [^3^].

Smartwatches and physical activity trackers have also been used to monitor physiological parameters, such as heart rate and body temperature, in addition to detecting falls and impacts, assessing noise exposure, and providing real-time feedback to workers. Studies have demonstrated that accelerometers incorporated into commercial smartwatches, despite technical limitations, can be used in occupational risk assessment applications [^4^,^5^]. In addition, the possibility of integrating these technologies into personal protective equipment, such as helmets, belts, and smart wristbands, has been highlighted, combining multiple sensors for comprehensive workplace safety monitoring. These multisensory platforms can trigger alarms upon anomaly detection and transmit data to centralized monitoring systems.

Corporate medical directors and occupational physicians have been evaluating AI applications across a spectrum ranging from rule-based systems to machine learning and deep learning/neural network models. Relevant applications in occupational health include: i) clinical decision support tools integrated into EHRs, capable of identifying abnormal results and suggesting therapeutic guidelines; ii) remote monitoring and management through prescribed digital therapeutics for conditions such as diabetes and substance use disorders; and iii) speech and image recognition technologies aimed at converting clinical interactions into medical documentation. In addition, predictive models enable the analysis of large data volumes, contributing to error reduction, increased efficiency, and support for clinical decision-making [^6^-^8^].

Although international studies have described promising AI applications in occupational health [^9^-^11^], there is a scarcity of studies focused on the reality of middle-income countries, such as Brazil, as well as on the perspectives of occupational physicians themselves as users of these technologies.

In this context, there is a knowledge gap regarding the degree of familiarity, perceived usefulness, and willingness to adopt advanced digital technologies and AI among Brazilian occupational physicians. This scenario may impact both the quality of workers’ health care and the efficiency of occupational health management [^12^].

Therefore, the aim of this study was to analyze the perceptions of Brazilian occupational physicians regarding the use of advanced digital technologies and AI in their professional practice.

## METHODS

This observational, cross-sectional study with a quantitative approach was conducted through an electronic survey. The target population consisted of occupational physicians registered in the communication database of the Brazilian National Association of Occupational Medicine (ANAMT).

The sample was composed of voluntary participants recruited through email invitations and dissemination via ANAMT institutional communication channels, characterizing a non-probabilistic sample. Approximately 1,600 physicians were invited to participate in the study, of whom 260 fully completed the questionnaire, corresponding to a response rate of 16.3%.

The data collection instrument consisted of a structured questionnaire developed based on theoretical constructs from the digital health and AI literature and previously submitted to pretesting to assess clarity and comprehension. The questionnaire included closed-ended questions, Likert-type scales, and multiple-choice questions.

Data collection was conducted using the Qualtrics XM Platform between May and June 2025. Participation was voluntary and conditioned upon acceptance of the informed consent form, in accordance with ethical principles applicable to research involving human subjects.

Data analysis was exclusively descriptive and included absolute and relative frequencies, measures of central tendency (mean and median), and measures of dispersion (standard deviation and 25th and 75th percentiles). Analyses were performed using Stata 19 (StataCorp LLC, College Station, T.X., USA).

The study was approved by a human research ethics committee under approval no. 116/2025.

## RESULTS

A total of 260 responses were obtained from a universe of 1,600 occupational physicians invited to participate in the study, corresponding to a 90% confidence level and a sampling margin of error of 4.69%.

The mean age of respondents was 51.7 years (standard deviation, 13.2 years), and 67.8% were male. Most had completed a specialization course in occupational medicine (184; 72.7%) and held a specialist certification in the field (174; 68.8%).

Regarding the professional practice setting, the private sector predominated (150; 58.4%), with full-time and exclusive professional practice reported by most participants (151; 58.5%) (Table 1).

**Table 1:** Workplace setting of occupational physicians

Workplace setting	Number	%
Private company	150	58.4
Consultancy/service provider company	46	17.9
Public service	30	11.7
Private practice	23	8.9
University (teaching and research)	8	3.1

Regarding the use of basic digital technologies, such as word processors, spreadsheets, and applications, most physicians reported using them both in their personal lives (238; 92.2%) and in the workplace (183; 70.9%). However, only 35.7% (92) had participated in courses or training related to digital technologies within the last 2 years.

In daily professional practice, occupational physicians who used technological resources reported employing EHR systems and clinical data recording tools (88%), telehealth tools (37%), business intelligence resources (30%), and clinical decision support systems focused on workers’ health care (20%).

Participants demonstrated varying levels of familiarity with advanced digital technologies applied to occupational medicine, with a mean score of 2.77 on a scale from 1 to 5 (Table 2).

**Table 2:** Degree of familiarity with advanced digital technologies in occupational medicine practice (scale: 1 [very low] to 5 [very high])

Degree of familiarity	Number	%
1	46	17.8
2	63	24.3
3	78	30.1
4	49	18.9
5	23	8.9

Overall, familiarity with advanced digital technologies was still predominantly moderate to low, which suggests an early stage in the incorporation of these tools into occupational medicine practice.

Most respondents reported using AI tools in the professional environment on a daily or weekly basis (52.1%), whereas approximately one-quarter stated that they never or rarely used them (Table 3).

**Table 3:** Frequency of artificial intelligence tool use in the professional environment

Frequency	Number	%
Daily	91	35.4
Weekly	43	16.7
Occasionally	58	22.6
Rarely	34	13.2
Never	31	12.1

Nearly half of the occupational physicians interviewed considered that advanced digital technologies contribute to improving workers’ health care, assigning scores of 4 or 5 on the evaluation scale (127; 48.6%) (Table 4).

**Table 4:** Perception of the contribution of advanced digital technologies to improving worker health care (scale: 1 [very low] to 5 [very high])

Scale	Number	%
No response	33	12.6
1	10	3.8
2	27	10.3
3	64	24.5
4	69	26.4
5	58	22.2

Respondents identified epidemiological data analysis of the working population (78%) and the automation of reports and medical evaluations (67%) as the areas of occupational medicine with the greatest potential for AI application (Table 5).

**Table 5:** Areas of occupational medicine with the greatest potential for artificial intelligence application

Areas	Number	%
Epidemiological data analysis of the working population	204	78
Automation of medical reports and documentation	173	67
Continuous worker health monitoring	161	62
Management of sick leave and return to work	145	56
Prevention of occupational accidents and diseases	100	38

These findings suggest that occupational physicians perceive greater AI potential in applications focused on population-level analysis and process automation compared with uses directly related to clinical care or prevention.

Finally, 234 respondents (91.1%) expressed willingness to participate in training programs on AI and digital technologies applied to occupational medicine, highlighting both interest in and the need for educational initiatives focused on the specific demands of the specialty.

## DISCUSSION

This study identified three main findings: i) still limited and heterogeneous use of advanced digital technologies among Brazilian occupational physicians; ii) perception of greater AI potential in applications focused on epidemiological analysis and process automation; and iii) high professional interest in training programs on the topic.

The predominantly low to moderate familiarity with advanced digital technologies suggests that AI incorporation into Brazilian occupational medicine is still at an early stage. This finding may be explained by gaps in medical education, especially among professionals with longer practice experience as well as by the limited availability of structured training throughout the professional career pathway.

Although more than half of respondents reported using AI tools on a daily or weekly basis, such use appears to be concentrated in administrative and informational support applications, such as report automation and data analysis, rather than in direct clinical support. This pattern suggests a pragmatic and incremental adoption of technology, focused more on operational efficiency than on transforming health care delivery.

The greater value attributed to AI in activities related to epidemiological analysis and process automation reflects structural characteristics of occupational medicine, which has traditionally been oriented toward population-based approaches, risk management, and the integration of multiple data sources. In this context, AI presents relevant potential for improving the analysis of large volumes of information derived from periodic examinations, sick leave records, occupational accidents, and organizational indicators, thereby supporting data-driven decision-making.

However, the international literature still provides limited evidence regarding the direct impact of AI on clinical and occupational outcomes, such as reductions in morbidity, mortality, or work absenteeism. Recent studies have described promising applications, including predictive return-to-work models and automated identification of psychosocial risk factors, while also emphasizing the need for greater methodological robustness and validation across different contexts.

Additionally, organizational and contextual factors appear to play an important role in the adoption of these technologies. Barriers such as lack of protected time, low institutional prioritization, and limited professional engagement have been described as important obstacles to AI incorporation into medical practice, particularly in settings with high clinical demand.

In the Brazilian context, these challenges may be even more pronounced, considering the heterogeneity of practice settings, differences in access to technologies, and the absence of specific guidelines for incorporating digital solutions into occupational health.

The high percentage of occupational physicians willing to participate in training programs constitutes a particularly relevant finding. This result suggests that the limited familiarity with advanced technologies does not stem from conceptual resistance, but rather from structural and educational barriers, indicating a window of opportunity for the development of training and continuing education strategies [^13^].

From an applied perspective, the results of this study reinforce the need for coordinated initiatives involving professional associations, educational institutions, and employer organizations to promote the development of competencies in digital health and AI within occupational medicine. Such initiatives may contribute to a more qualified incorporation of these technologies, with potential impacts on service efficiency, quality of care, and management of workers’ health [^4^,^14^].

One limitation of the present study relates to the non-probabilistic nature of the sample and the voluntary participation of respondents, factors that may introduce selection bias and limit the generalizability of the results. Furthermore, because this was a cross-sectional study, it is not possible to establish causal relationships between the variables analyzed.

## CONCLUSIONS

The results of this study indicate that, although Brazilian occupational physicians widely use basic digital technologies, the incorporation of advanced digital technologies and AI tools still occurs in a limited and heterogeneous manner. Greater familiarity with and appreciation of AI were observed in applications focused on epidemiological analysis of the working population and automation of reports and processes, reflecting the collective and managerial logic that characterizes occupational medicine practice.

The predominantly low to moderate familiarity with these technologies contrasts with the high level of professional interest in participating in training programs, suggesting that the main barriers to AI adoption are not related to conceptual resistance, but rather to insufficient specific training and the scarcity of structured educational opportunities.

These findings reinforce the need for institutional strategies aimed at developing competencies in digital health and AI applied to occupational medicine, involving professional associations, specialization programs, and employer organizations.

Future studies should advance toward comparative and longitudinal analyses, evaluating factors associated with technological adoption and the impact of these tools on health care, organizational, and workers’ health outcomes.

Understanding this scenario is essential to support training strategies and technological incorporation into occupational medicine practice in Brazil.

## Data Availability

The data supporting the findings of this study are available within the article
